# A micro-patterned transient UV photodetector enabled by solvent-free microfabrication

**DOI:** 10.1038/s41378-025-01012-3

**Published:** 2025-08-20

**Authors:** Zhiqing Xu, Qinhua Guo, Lizhou Yang, Jiajun Zhang, Xiwen Liu, Qinghao He, Man Chan, Yunda Wang

**Affiliations:** The Hong Kong University of Science and Technology (Guangzhou), No.1 Du Xue Rd, Nansha District, 511544 Guangzhou China

**Keywords:** Optical sensors, Optical physics

## Abstract

Transient electronics, which can be controllably broken down with zero environmental impact, hold significant potential in implantable devices, hardware security, and disposable sensors. While miniaturization is essential for enhancing device performance, increasing integration density, and enabling new applications, degradable materials often face challenges with conventional microfabrication processes like lithography due to their sensitivity to heat and solvents. In this paper, we present a UV photodetector (PD) with micro-scale patterning, fabricated using a novel solvent-free material patterning method. The PD, consisting of molybdenum (Mo) as the electrode, zinc oxide (ZnO) as the photoactive material, and polyvinyl alcohol (PVA) as the substrate, can be dissolved in deionized (DI) water, leaving behind non-toxic byproducts. The device exhibits high responsivity over 50 A/W and an obvious response to varying sunlight intensities, demonstrating its potential for temporary, eco-friendly UV sensing applications. Additionally, we demonstrated that the photoresist used in the solvent-free material patterning method can be reused for subsequent fabrication while maintaining good registration, enhancing efficiency and reducing material waste. This approach provides a scalable and high-efficiency microfabrication strategy for integrating functional materials into unconventional platforms, offering broader applicability in next-generation transient, biodegradable, and flexible sensor technologies.

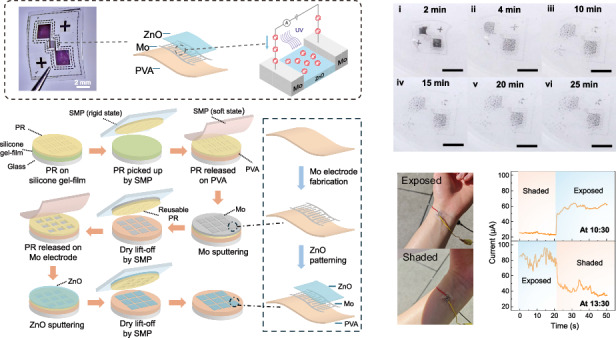

## Introduction

Modern electronic devices are designed to be mechanically robust, chemically stable, and durable for long-term use. Transient electronics fabricated by degradable materials, on the contrary, allow materials, components, and circuits to break down in a controlled manner, leaving minimal or zero environmental impact^1,2^. Such unique characteristics open up opportunities for wide-range applications including implantable medical devices^3–5^, hardware security^6^, wearable and disposable sensor^7,8^. Implantable bioresorbable devices, for instance, naturally degrade within the body, eliminating the need for secondary surgeries^9^, while hardware-secured electronics can disintegrate on demand to protect sensitive information^10^. Disposable polymer-based sensors offer affordable short-term monitoring^11^ but contribute to increasing global electronic waste, as only 17.4% being properly recycled for 2019^12^. Eco-friendly transient electronics thus represent a critical step toward sustainable and disposable technologies^13^.

Disposable and flexible ultraviolet (UV) photodetectors (PDs) play a crucial role in various applications, including hazardous UV detection, wearable dosimetry, secure communication, and health and environmental monitoring^8^. Researchers have also explored degradable UV PDs for eco-friendly green electronics. For example, Yalagala et al. reported a high-performance UV photodetector using ZnO nanowires on a flexible chitosan substrate, showcasing excellent biodegradability and photoresponsivity^8^. However, integrating these materials into micro-scale devices remains challenging. Traditional microfabrication processes, such as lithography, often involve high temperatures and chemical solvents that can degrade or damage these sensitive substrates^14^. While direct microfabrication methods on degradable substrates have been explored, such as the lithography-based process on protected polylactic acid (PLA) substrates^15^ and the ferroelectric lithography on semicrystalline PLA films^16^, these approaches have limitations, including incompatibility with water-soluble substrates and material-specific constraints.

In this study, we present a UV photodetector with micro-scale patterning, fabricated by a solvent-free material patterning method. The device, constructed with molybdenum (Mo) electrodes, zinc oxide (ZnO) as the photoactive material, and polyvinyl alcohol (PVA) as a substrate, demonstrate excellent solubility in deionized (DI) water, leaving non-toxic residues. The UV PD exhibits high optical responsiveness to both UV and visible sunlight, demonstrating potential for transient, environmentally friendly sensing applications such as disposable and wearable outdoor UV monitoring. This work is expanded from our previous study^17^, presenting systematic study in terms of device mechanism, comprehensive characterization, demonstration on photoresist reuse, and investigation on the effect of electrode design on device’s performance.

During device fabrication, we successfully demonstrate micro-scale (10 μm) patterning on a water-soluble PVA substrate using our solvent-free method. Additionally, we show that the photoresist employed in the patterning process can be reused multiple times while maintaining good registration accuracy. This approach significantly enhances fabrication efficiency, reduces material waste, and holds great potential for broader applications in sustainable and transient electronic systems.

## Result and discussion

**Fig. 1:**
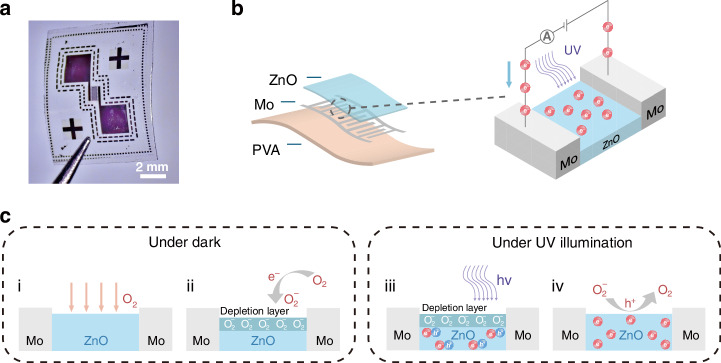
Device structure and operating mechanism. **a** Optical image of the fabricated UVPD. **b** Schematic illustration of the device structure and schematic of photocurrent detection. **c** Mechanism of photoelectron generation by absorption and desorption of oxygen

Figure 1 shows the optical image of the UV PD and the mechanism of photocurrent detection. Figure 1a shows the optical image of the device. Figure 1b illustrates the device structure. The device consists of ZnO as photoactive material, Mo as the electrode material, and PVA as the substrate, which are all environmentally friendly materials. The enlarged view of the schematic illustrates the working principle of the device. Under UV illumination, ZnO absorbs photons with energy greater than its bandgap, generating electron–hole (e–h) pairs. The photogenerated electrons are conducted through the Mo interdigital electrodes, forming a photocurrent. Figure 1c illustrates a mechanism of photoelectron generation. Other than increasing number of photoactivated electron–hole (e–h) pairs, oxygen absorption and desorption process also play an important role in photoelectron generation of ZnO-based PD^18^. The mechanism can be understood by the following steps: (i) during the dark condition, the oxygen molecules (O_2_) from the air atmosphere get adsorbed by the surface of ZnO layers. (ii) The adsorbed oxygen (O_2_) has the tendency to react with free electrons (e−) and forms depletion region. (iii) When ZnO thin films are illuminated by UV light greater than the bandgap of ZnO thin film, the electron–hole (e–h) pairs are generated. (iv) The photogenerated carriers reach the ZnO surface and neutralize the adsorbed oxygen. The depletion layer formed by negatively charged oxygen ions results in upward band bending near the surface. With UV illumination, electron–hole pairs are generated with light of energy exceeding the bandgap. The photogenerated holes migrate along the bent bands and react with the negatively charged oxygen ions, while the photogenerated electrons enhance conductivity by increasing carrier concentration and reducing the potential barrier near the oxygen adsorption sites^19^.

### Device fabrication

**Fig. 2:**
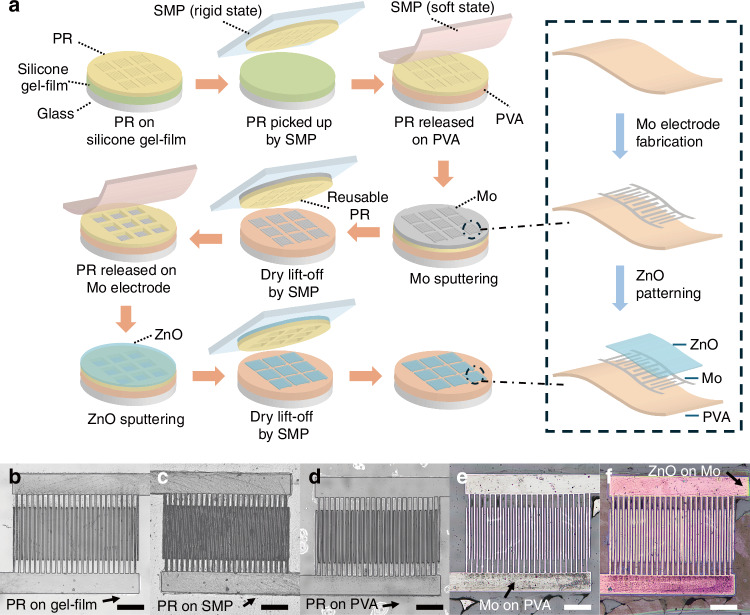
Fabrication process of the water-soluble photodetector using a solvent-free material patterning technique. **a** Schematic illustration of the solvent-free material patterning process enabled by photoresist (PR) dry transfer printing using an SMP stamp. **b** Patterned PR on a gel-film substrate. **c** PR picked up by the SMP. **d** PR released onto a PVA substrate. **e** Mo electrodes on the PVA substrate after Mo sputtering and PR removal. **f** ZnO patterned on Mo electrodes following ZnO deposition and PR removal. Scale bar: 200 μm

Figure 2 illustrates the device fabrication process of the solvent-free material patterning process used in this study. The process is based on a transfer printing technique that utilizes a thermo-responsive SMP, which undergoes a sharp modulus change around its glass transition temperature^20^. By changing temperature, SMP can switch between rigid state and soft state, which are responsible for PR locking and releasing, respectively. The detailed mechanism of PR transfer printing has been previously reported^20^. Figure 2a shows the overall fabrication process. First, a patterned PR layer is transferred from a silicone gel-film/glass substrate onto a PVA substrate using SMP as the transfer medium. The SMP, heated to its soft state, is stamped and pressed onto the PR. Upon cooling, it hardens and forms a strong adhesion to the PR. The SMP is then peeled off, carrying the PR, and subsequently reheated to release the PR onto the PVA due to its low adhesion in the soft state. This process successfully transfers the PR onto the PVA substrate. In the process, the PR is patterned on a silicone gel film (with a thickness of 6.5 mil), which is selected due to its favorable combination of surface energy and mechanical compliance. This combination provides a relatively low critical energy release rate during the peeling step, making it easier for the PR to be released from the gel-film and transferred to the target substrate. However, if the surface energy is too low, it can compromise the adhesion during the initial photolithography step. To address this, a short oxygen plasma treatment is applied to the silicone gel film surface to enhance its adhesion for high-resolution patterning. At the same time, no adhesion promoter is used in the lithography process to avoid excessive adhesion that could hinder successful PR release. Under these optimized conditions, the locking force of the SMP stamp, when cooled to its rigid state, is sufficient to ensure reliable PR pickup and transfer.

After the PR transfer, a thin Mo layer is then sputtered onto the PVA with the SU-8 PR pattern. Instead of using solvents for conventional lift-off, a “dry lift-off”^17^ process is employed, where the SMP is used to peel off the PR, leaving behind the patterned metal electrodes. This solvent-free method functions similarly to traditional lift-off but eliminates chemical exposure and enables PR reuse. The removed PR remains intact and can be transferred onto another substrate for additional fabrication cycles. Using the same transfer approach, PR for the semiconductor layer is aligned with the electrodes and transferred, followed by the deposition of a ZnO layer to complete the device. Figure 2b–f shows optical images of different stages in the fabrication of a device using this method.

Figure 2b shows PR is patterned on silicone gel-film, which is then picked up by SMP (Fig. 2c) and released on PVA substrate (Fig. 2d). After Mo sputtering and PR removal, Mo electrode is fabricated on PVA substrate, as shown in Fig. 2e. With the same process, microscale ZnO is fabricated on interdigital electrode area. Figure 2f presents the completed device after ZnO deposition. The fabricated device consists of a 58-nm-thick Mo electrode layer and a 343-nm-thick ZnO layer, while the patterned PR used in the process has a thickness of 10 μm. The detail parameter is illustrated in materials and methods section.

### PR reuse demonstration

**Fig. 3:**
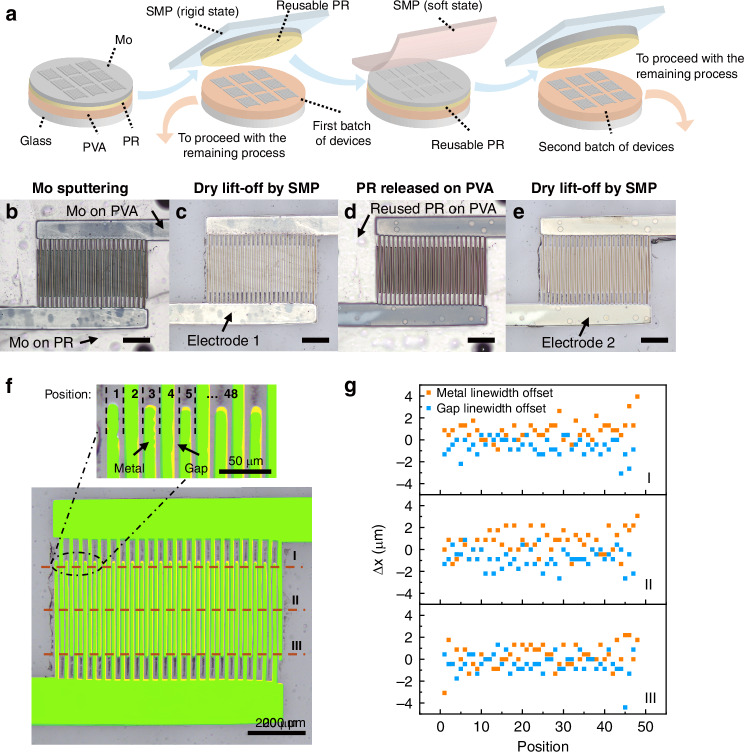
Fabrication and transfer registration error of reusable PR. **a** Schematic illustration of PR reuse process. **b** PR on a PVA substrate with Mo deposition. **c** Mo electrode on the PVA substrate after PR removal, referred to as “Electrode 1”. **d** PR removed from the PVA substrate in **c** is released on another piece of PVA substrate. **e** Mo electrodes fabricated using the reused PR, referred to as “Electrode 2”. **f** Overlapping image of Electrode 1 (yellow) and Electrode 2 (green). The interdigital region is divided into 48 sections, each containing a metal and gap area. **g** Linewidth difference (*Δx*) of electrode 2 and electrode 1 in region I, II, and III respectively. (scale bar in **b**–**e**: 200 μm)

The PR removed by the “dry lift-off” process remains well-preserved on the SMP stamp and can be reused on another PVA substrate for subsequent fabrication. Figure 3 shows microscope images illustrating the PR reuse process and the corresponding registration accuracy of the electrodes fabricated using the same PR. Figure 3a shows the PR reuse process, with the blue arrows indicating the flow of the same PR. After Mo sputtering (Fig. 3b), PR is removed via “dry lift-off”, leaving the electrodes on PVA substrate, named as electrode 1 (first fabrication batch, Fig. 3c) which is the first batch of fabrication. The removed PR is then transferred onto a second PVA substrate (Fig. 3d) for the next fabrication batch, forming electrode 2 (Fig. 3e). Figure 3f shows the overlapped processed image of electrode 1 (yellow) and electrode 2 (green). The interdigital finger region is divided into 48 sections, each of which consists of metal and gap areas. Metal areas from electrode 1 and electrode 2 at position 1 align precisely in the overlapped region. The linewidths of metal and gap areas are measured in three regions (I, II, and III) on both electrodes. Registration error is analyzed by calculating the linewidth difference of two electrodes. The linewidth difference is defined by Eq. (1):

1 $$\Delta x={x}_{2}-{x}_{1}$$

Where *x*_1_ is linewidth of electrode 1, *x*_2_ is linewidth of electrode 2, Δ*x* is the linewidth difference.

Figure 3g shows the differences in metal and gap linewidths between electrode 2 and electrode 1 in each region. A negative offset value indicates that the corresponding metal or gap linewidth in electrode 2 is narrower than that in electrode 1, and vice versa.

As shown in Fig. 3g, the measured offsets for both the metal electrode width and the gap width are mostly within ±2 µm, indicating high registration accuracy. Considering the measurement uncertainty of ±2.9 µm, these offsets remain within a narrow range. The linewidth difference is likely due to PR deformation during transfer, which has a more obvious effect on small-linewidth PR area. Offset in region II is generally larger than the other two regions. It is because region I and region III are located at the two ends of the metal area, supported by larger-area PR to prevent deformation. It can also be observed in Fig. 3f that metal area near position 1 and position 48 is better overlapped compared to the middle area. It is also the result of adjunct large-area PR. Detailed measurement methods and uncertainty calculations are provided in the Supplementary Information. The demonstration of reusable photoresist with relatively low registration error shows the fabrication process has the advantage of low waste and high fabrication efficiency. The registration error measurement is not performed on ZnO patterning process in this work, as the ZnO region (~600 µm × 960 µm) is significantly larger than the electrode patterns and tolerates a greater registration error. Therefore, high-precision alignment was not critical for that step. For ZnO patterning with thickness over 300 nm, profile distortions may arise from two aspects. One is PR deformation caused by repeated mechanical stress during transfer. The other is the accumulation of ZnO on the PR sidewalls during sputtering, which can narrow openings and round corners, leading to a loss of pattern fidelity over repeated reuse cycles. These factors may influence pattern accuracy and should be carefully considered when designing processes that balance alignment tolerance and the number of reuse cycles.

### Material characterization

**Fig. 4:**
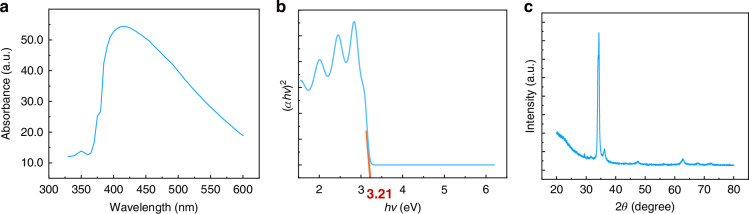
ZnO thin film characterization. **a** Absorbance of ZnO thin film. **b** Bandgap of ZnO calculated with absorbance. **c** XRD spectra of ZnO thin film

To understand the optical and structural properties of the ZnO thin film, we performed a series of characterizations. As shown in Fig. 4a the absorbance spectrum, with a maximum absorption peak at 410 nm, confirming ZnO’s strong UV absorption capability. Figure 4b shows the Tauc plot derived from the absorbance data to determine the band gap energy. The band gap of ZnO thin film is calculated using the Tauc equation (Eq. (2))^21^:

2 $${\left(\alpha {hv}\right)}^{\displaystyle\frac{1}{n}}={A}^{* }\left({hv}-{E}_{g}\right)$$

where *hv* is the energy, *α* is the absorption coefficient, *E*_*g*_ is the band gap, the value of *n* is considered as 0.5 for the direct band gap materials and A^*^ is the slope of the Tauc plot in the linear region. and the slope of the plot (*αhv*)^2^ vs (*hv*) gives the direct band gap of the ZnO thin film. The calculated band gap energy of the ZnO is 3.21 eV, consistent with the previously reported value^22,23^. Figure 4c shows the X-ray diffraction (XRD) spectrum of the ZnO thin film, where diffraction peaks at 2θ angles of 34.3°, 36.2°, 47.5°, and 62.8° correspond to the (002), (101), (102), and (103) crystal planes, respectively^8^. This indicates a well-ordered crystalline structure, which contributes to better performance of the device^24^.

### Device characterization

**Fig. 5:**
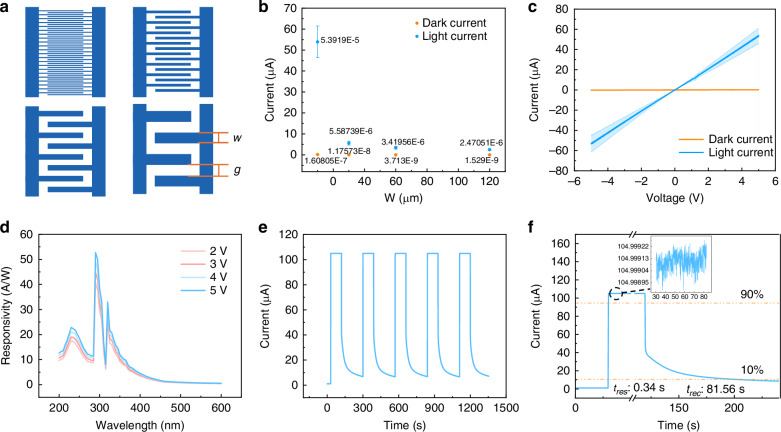
Photoelectrical characterization of PD. **a** Designs of electrodes with different linewidths of interdigital finger. **b** Comparison of device with electrodes linewidth of 10 μm, 30 μm, 60 μm, and 120 μm with 5-V voltage under dark condition and UV illumination of 46.9 mW/cm². The experiment is repeated three times. **c** Current of the 10-μm-design device under dark condition and UV illumination with intensity of 47.3 mW/cm^2^. The experiment is repeated three times. **d** Responsivity of the same 10-μm-design device under bias voltage of 2 V, 3 V, 4 V, and 5 V. **e** Temporal respond current of the same device under dark condition and UV illumination with intensity of 227.6 mW/cm^2^. **f** Single cycle of temporal respond. Response and recovery time are calculated by current changing from 90% of maximum current to 10% of maximum current

Devices with different interdigitated electrode designs are fabricated to compare their light and dark current characteristics. Supplementary Fig. S2 illustrates the electrode layouts and ZnO pattern. While the interdigital finger width varies, the exposed active sensing area and total metal area remain consistent across all the designs. The linewidth of the interdigital finger is defined as *w* in the schematic of Fig. 5a. For each design, the gap between adjacent fingers is defined as *g*, where *w* = *g*. The four designs have *w* = *g* = 10 μm, 30 μm, 60 μm, and 120 μm, respectively. All devices share the same ZnO patterning layout.

Figure 5b shows the light and dark current of each device under a 5-V bias voltage. The light current is measured under 365 nm UV illumination with an intensity of 46.9 mW/cm², while the dark current is recorded with the devices covered by tinfoil to block ambient light. Each experiment is repeated three times for consistency. The results indicate that as *w* decreases, both dark and light currents increase, with the 10-μm design showing the highest values. The larger light current enhances detectability, demonstrating the benefit of high-resolution fabrication. The observed increase in current with decreasing interdigital electrode width can be understood from two complementary perspectives. From a carrier transport perspective, the narrower gap between electrode shortens the path that photogenerated electrons need to travel before reaching the metal electrodes, reducing electron recombination rate. Therefore, it contributes to larger photocurrent. From an electrical modeling perspective, the ZnO region between the electrodes can be viewed as a resistive medium, and the electrode structure can be approximated as a parallel resistor network. When the total sensing area and metal coverage are fixed, reducing the gap width increases the number of conductive paths and lowers the overall resistance, resulting in greater current under illumination. The detail of calculation is presented in Supplementary Information.

Detailed characterization is conducted on the device with *w* = 10 μm. Figure 5c–f shows the characterization results from the same device. Figure 5c shows the current-voltage (I–V) characteristics of the device under dark conditions and UV illumination at 47.3 mW/cm² with a voltage sweeping from −5 V to +5 V. Each experiment is repeated three times for consistency. The linear I–V characteristics suggest that the ZnO/Mo interface exhibits Ohmic contact behavior^25^. While ZnO and Mo typically form a Schottky contact, the observed Ohmic contact can be attributed to ion bombardment during Ar plasma treatment, which reduces the contact barrier^26^. The ohmic contact may also result from other factors such as interface conditions, material purity, or defect states, which can reduce the barrier height.

Responsivity (*R*_*λ*_) is an important parameter to evaluate the performance of photodetector, which indicates how efficiently a detector responds to optical signals^27^. The responsivity of the same device with *w* = 10 μm is derived from the measured external quantum efficiency (EQE) using Eq. (3):

3 $${R}_{\lambda }=\frac{{e}_{\lambda }}{{hc}}{EQE}$$

Where λ is the exciting wavelength, *h* is the Planck’s constant, *c* is the velocity of light, and *e* is the electronic charge. The EQE values were experimentally obtained as described in the Methods section.

Figure 5d shows the responsivity spectra of the device under bias voltages of 2 V, 3 V, 4 V, and 5 V. The highest responsivity peak occurs at ~290 nm, with additional peaks observed at 230 nm and 320 nm. At 290 nm, the photodetector achieves a high responsivity of over 50 A/W under a 5-V voltage. Figure 5e shows the time-dependent photoresponse of the device applied with voltage of 5 V. Current is measured with the 365 nm UV illumination of 227.6 mW/cm^2^ for 90 s and turning off for 180 s, which is recorded by a source meter with sampling interval of 0.08 s. The result shows the device provides stable and cyclic photoresponse. Figure 5f provides an enlarged view of single time-dependent on/off response cycle. The measured response time (*t*_*res*_) and recovery time (*t*_*rec*_) are shown in Fig. 5f. The device rises quickly in a short *t*_*res*_ of 0.34 s and then decays *t*_*rec*_ within 81.56 s, defined as the time intervals between 10% and 90% of the saturated photocurrent amplitude during rise and decay, respectively. The quick rise time is contributed by the rapid formation of photogenerated electron and hole (e–h) pairs (*hv* → *e* + *h*). The slow process of recovery is due to the absorption and desorption of oxygen which dominates the recovery process and require relatively longer time^19^. Similar result is also observed from other ZnO-based photodetectors^18,28^.

**Fig. 6:**
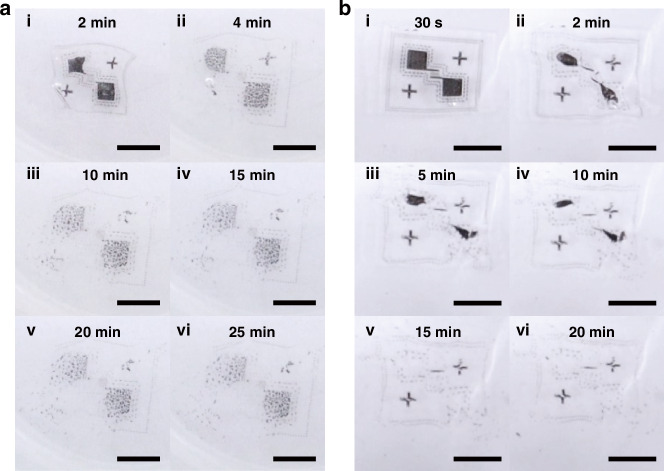
Photographs of devices in DI water of room temperature and 70 °C, taken at different time intervals. **a** Optical images of the device immersed in DI water of room temperature after 2 min, 5 min, 10 min, 15 min, 20 min, and 25 min. The device is 108-μm thick with an area of 9.78 mm × 8.67 mm. **b** Optical images of the device immersed in DI water of 70 °C after 30 s, 1 min, 2 min, 5 min, 15 min, and 20 min. The device is 115-μm thick with an area of 9.58 mm × 7.97 mm. (scale bar: 5 mm)

A solubility test was conducted on devices in DI water in both room temperature and 70 °C. The device was immersed in the center of a 150-mL beaker containing ~45 mL of DI water. Figure 6a shows optical images of a device immersed in room-temperature DI water at different time intervals: 2 min, 5 min, 10 min, 15 min, 20 min, and 25 min. The device, measuring 108 μm in thickness with a rectangular area of 9.78 mm × 8.67 mm, undergoes gradual dissolution as the PVA substrate dissolves. This process leads to the disintegration of metal electrodes, and by 25 min, the device is fully dissolved, with Mo and ZnO particles dispersed in the water.

A similar test was conducted on another device with comparable thickness and size in DI water at 70 °C. This device is 115-μm thick with a measured rectangular size of 9.58 mm × 7.97 mm. Figure 6b shows optical images captured at different time intervals: 30 s, 1 min, 2 min, 5 min, 15 min, and 20 min. The results indicate that the device degrades faster in hot water. By 20 min, it is fully dissolved, leaving only dispersed metal particles in the water.

**Fig. 7:**
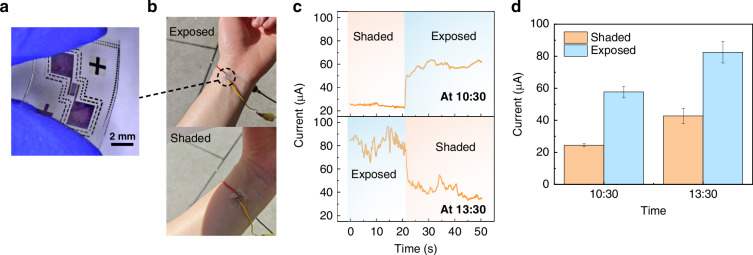
Application of PD in outdoor environment. **a** Optical image of the device. **b** Image of the device on arm in exposed and shaded conditions. **c** Continuous measurement of current with 5-V voltage under exposed and shaded states at ~10:30 and ~13:30 respectively. **d** Average current of the device in exposed and shaded conditions applied with 5-V voltage tested at ~10:30 and ~13:30 respectively. The error bar in the bar graph is calculated by mean and standard deviation of the data from (**c**)

To evaluate the feasibility of the device for wearable UV detection, a demonstration experiment was conducted outdoors. As shown in Fig. 7b, a device was placed on a user’s arm, directly exposed to sunlight. To assess its response under different outdoor lighting conditions, the current was measured when the device was directly exposed to sunlight and when placed in a shaded area created by a standard A4 paper sheet. The measurements were taken at 10:30 AM and 1:30 PM on the same day and at the same location, with stronger sunlight expected around noon. The result, presented in Fig. 7c, shows the device has a fast and obvious response when shifted between shaded and exposed states. Figure 7d demonstrates that the device effectively detects variations in UV intensity, producing higher currents under stronger sunlight conditions. The error bar is calculated by mean and standard deviation of the continuous current from Fig. 7c.

## Conclusion

We have developed a water-soluble, environmentally friendly UV photodetector using a solvent-free material patterning method based on photoresist transfer. The fabricated device exhibits high responsivity, stable on-off switching characteristics, and rapid degradability in DI water, leaving only non-toxic residues. These features make it highly promising for wearable transient electronic applications. A key contribution of this work is the solvent-free micro-patterning technique using shape memory polymer (SMP) as a transfer stamp, achieving high-resolution fabrication (10 μm) on water-sensitive PVA substrates. This method prevents solvent-induced damage on substrate and enables repeated photoresist reuse with excellent registration accuracy (within ±2 μm), offering significant advantages including low waste, high fabrication efficiency, and compatibility with unconventional substrates. The presented approach can be easily adapted for fabricating electronics on other unconventional materials, expanding potential applications in biodegradable, flexible, and transient electronics.

## Materials and methods

### Overview of the fabrication process

To fabricate the device, a 10-μm-thick SU-8 2010 photoresist is first patterned on a silicone gel-film/glass wafer substrate which is picked up and transferred to PVA by SMP. SMP heated to soft state is stamped and pressed onto the SU-8 PR to conformally contact. The SMP turns into rigid state with strong adhesion after cooling, locking the embedded SU-8 PR in place. It is then peeled away and carries the SU-8 PR. Next, the SMP is reheated and returned to its soft state and placed onto a PVA substrate. The SMP is then slowly peeled off, releasing the SU-8 PR onto the PVA due to its low adhesion.

A 58-nm-thick Mo layer is sputtered onto the PVA with the SU-8 PR pattern. The SU-8 PR is removed using the SMP again with a “dry lift-off” process, leaving the metal layer patterned. As a result, interdigital electrodes are fabricated on the PVA. The peeled off PR is well-preserved and can be released on another PVA substrate to undergo another batch of fabrication. Using the same transfer process, PR for ZnO patterning is picked up from silicone gel-film substrate, which is aligned with the electrode and released on it. A 343-nm-thick ZnO layer is fabricated on top of the interdigitated electrodes. The materials and parameters are detailed in the following sections.

### Lithography on silicone gel-film

The commercial silicone gel-film is cut into 1 cm × 1 cm pieces and fixed onto a glass substrate using tweezers. To enhance surface wettability, the substrate is treated with oxygen plasma at 40 W for 30 s using a CIF plasma cleaner. SU-8 2010 is then spin-coated at 3000 rpm for 30 s followed by baking at 65 °C for 60 s and 100 °C for 270 s. It is then exposed to 365 nm UV light at an intensity of 16.4 mW/cm² for 8.5 s, followed by post-exposure baking is then performed at 65 °C for 30 s and 100 °C for 270 s. Finally, the 10-μm-thick SU-8 2010 photoresist is developed in the SU-8 developer for ~12 s.

### Preparation of PVA film

Two grams of PVA powder are dissolved in 20 mL deionized (DI) water with magnetic stirring at 130 °C. The PVA solution is poured into a clean plastic petri dish and degassed in a vacuum chamber, to remove trapped air bubbles, and the dried in an oven at 55 °C, resulting PVA film with thickness of ~110 μm. The PVA film is peeled off and cut into 1.5 cm × 2.5 cm pieces. A piece of film is then flattened with tweezers and fixed onto a glass substrate using PI tape.

### Solvent-free photoresist transfer printing using SMP

A thermo-responsive SMP is used as stamp in the solvent-free photoresist transfer process. It is fabricated by mixing 80 parts stearyl acrylate (SA), 20 parts urethane diacrylate (UDA), 1 part trimethylolpropane triacrylate (TMPTA), 1 part 2,2-dimethoxy-2-phenyl-acetophenone (DMPA), and 0.5 parts benzophenone (BP) at 60 °C, followed by UV curing. The same material preparation process and modulus transition of the SMP, from 145.8 kPa (soft) to 219.6 MPa (rigid) around its glass transition temperature (T_g_) of ~44 °C, have been previously reported^29^.

The transfer process consists of two steps: pick-up and release. To pick up SU-8 PR from the silicone gel-film surface, the SMP is stamped onto the SU-8 PR and slightly pressed to make good contact while heated on a hotplate set to ~80 °C. At this temperature, the SMP is in its soft state, facilitating good contact with the SU-8 PR. Upon cooling to room temperature, the SMP becomes rigid, embedding the SU-8 PR. It is then peeled away at a fracture propagation speed of ~0.3 mm/s, picking up the SU-8 PR. To release the SU-8 PR onto the PVA, the SMP is reheated on a hotplate set to ~80 °C to return to its soft state. Both the PVA and SU-8 PR are stacked on the same 80 °C hotplate to make the SMP remains soft during contact. The SMP is then slowly peeled off at a fracture propagation speed of ~0.17 mm/s, transferring the SU-8 PR onto the PVA. The propagation speeds are estimated from videos that record the processes. After Mo deposition, the SU-8 PR is picked up with the SMP using the same pick-up process described earlier, effectively performing a “dry lift-off” while maintaining the photoresist intact for reusing, which leaves the interdigital electrode pattern on the PVA. Next, another layer of SU-8 PR for ZnO patterning is aligned under microscope and transferred onto the electrode region on the PVA. A ZnO layer is then deposited using magnetron sputtering. The SU-8 PR is subsequently removed using the same pick-up process with the SMP stamp, leaving the ZnO layer patterned on the PVA.

### Thin-film deposition process

*Mo sputtering:* The Mo layer is deposited onto PVA using DC magnetron sputtering at room temperature. A Mo target with purity of 99.95% is used for deposition. Before deposition, the sample is cleaned by argon plasma with flow rate of 20 sccm for 2 minutes. The chamber base pressure is evacuated to 2 ×10^−6 ^Torr. The target is pre-sputtered for 30 s to eliminate contaminants on the surface. Mo is sputtered twice in an argon atmosphere at 10 sccm flow rate and 50 W power, with each sputtering step lasting 5 min and separated by a 5-min cooling interval. The cooling step is necessary to minimize thermal stress in the thin film, which can occur due to heating of the PVA substrate during the deposition process.

*ZnO sputtering:* A ZnO ceramic target with purity of 99.99% is used for deposition at room temperature. Before deposition, the sample is cleaned with argon plasma at a flow rate of 20 sccm for 2 min. The base pressure of the chamber is evacuated to 2× 10^−6 ^Torr. The target is pre-sputtered for 30 s to remove contaminants. ZnO is sputtered in an argon atmosphere at 20 sccm and 100 W for three cycles of 10 min each, with 10-min cooling intervals between cycles to prevent issues caused by heating of the PVA substrate, such as deformation.

### PR reuse

After Mo sputtering, PR is removed by SMP through “dry lift-off”. The PR on SMP is then released on PVA substrate using the method illustrated above. It is then sputtered with Mo following by “dry lift-off” for PR removal.

### Characterization

XRD of ZnO thin film is recored by Empyrean 3.0. Absorbance is measured by the spectrophotometer of LAMBDA 1050+. Thin film thickness is measured using a step profilometer (Bruker Dektakxt). Spectral response is measured by CROWNTECH QTest HIFINITY 5 and Keithley 2400 source meter. Light current and dark current are measured with DC source meter (GW Instek GSM-20H10) applied with changing voltage and illumination of 365-nm UV light. On-off switching properties are measured by Keithley 2450 source meter with 5-V bias voltage with illumination of a UV light. Two different UV light sources are used in the photoelectrical characterizations due to availability and experimental flexibility. A UV chamber (Seoul Viosys UV 1000-36) is used for steady-state measurements of I–V characterization, while a portable UV lamp is used for the on/off switching tests to allow more flexible operation. The emission spectrum of the UV lamp is provided in Supplementary Fig. S4. The emission spectrum of the UV chamber is available from the manufacturer’s product handbook, which confirms a similar emission spectrum with the UV lamp including peak around 365 nm, full width at half maximum, and shape of spectrum. The solubility of the device is tested in DI water at room temperature and 70 °C. Water temperature is measured by the thermometer probe of hotplate. Application in outdoor environment is measured by placing the device on arm while connecting to source meter (GW Instek GSM-20H10). The current is measured when the device is exposed to sunlight and shaded respectively at different time period and same location.

### Material used in the experiment

The SU-8 2010 photoresist and SU-8 developer is purchased from MicroChem, America. The Polyvinyl alcohol powder (degree of hydrolysis: 87~89 mol%, viscosity: 54–66 mPa) is acquired from Macklin Biochemical Technology Co., Ltd, China. The silicone gel-film (PF-30×30-0065-X4) with a rated thickness of 6.5 mil is obtained from GelPak, America.

## Supplementary information

supplementary information

device dissolved in DI water of room temperature

device dissolved in DI water of 70 degree C
