# Supplementary material for: A micro-patterned transient UV photodetector enabled by solvent-free microfabrication
Source: Microsyst Nanoeng. 2025 Aug 20;11:162. doi: 10.1038/s41378-025-01012-3 (PMC12368088; doi:10.1038/s41378-025-01012-3)
Supplement: Supplementary file 1 — supplementary information [file 41378_2025_1012_MOESM1_ESM.docx]

***Supplementary Material***

A Water-Soluble and Eco-Friendly UV Photodetector with a Solvent-Free Micro-Patterning Process

Zhiqing Xu^1^, Qinhua Guo^1^, Lizhou Yang^1^, Jiajun Zhang^1^, Xiwen Liu^1^, Qinghao He^1^, Man Chan^1^, and Yunda Wang^1*^

^1^The Hong Kong University of Science and Technology (Guangzhou), Guangzhou, CHINA

^*^Corresponding author. Email: ydwang@ust.hk

Uncertainty of linewidth measurement

The linewidth is measured by the optical image with 2.0728 pixels/μm, each pixel represents 0.482 μm. The uncertainty of measurement comes from the error of pixel selection on the edge of metal and gap area. Fig. S1 shows the edge of metal area across 6 pixels, showing the uncertainty of measurement is ±3 pixels, which is ±1.446 μm. The uncertainty of linewidth difference *Δx* is ±2.892 μm.


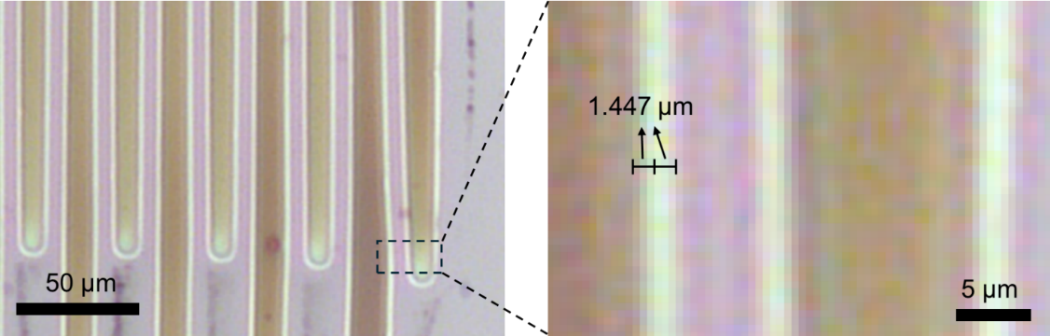


**Fig. S1 Enlarged image of the electrode.**

Layout designs of electrodes and ZnO patterning

Fig. S2 shows the layout designs of electrodes and ZnO patterning. Fig. S2a-d shows electrode designs with interdigital linewidth of 10 μm, 30 μm, 60 μm, and 120 μm respectively. Fig. S2e shows the layout of ZnO patterning. The large alignment mark is for photoresist alignment. The dash-line pattern surrounding the electrode design is to protect the major area from cracking during transfer. Fig. S3 shows the transfer process^1^. When rubbery SMP conformably contacts the PR, SMP forms “claws” in the patterned area. After cooling, the SMP grips up the PR when peeling away. Dash-line patterns provide more “gripping points” to facilitate the transfer process. Fig. S4 shows the emission spectrum of the UV light source used in the on-off response characterization.


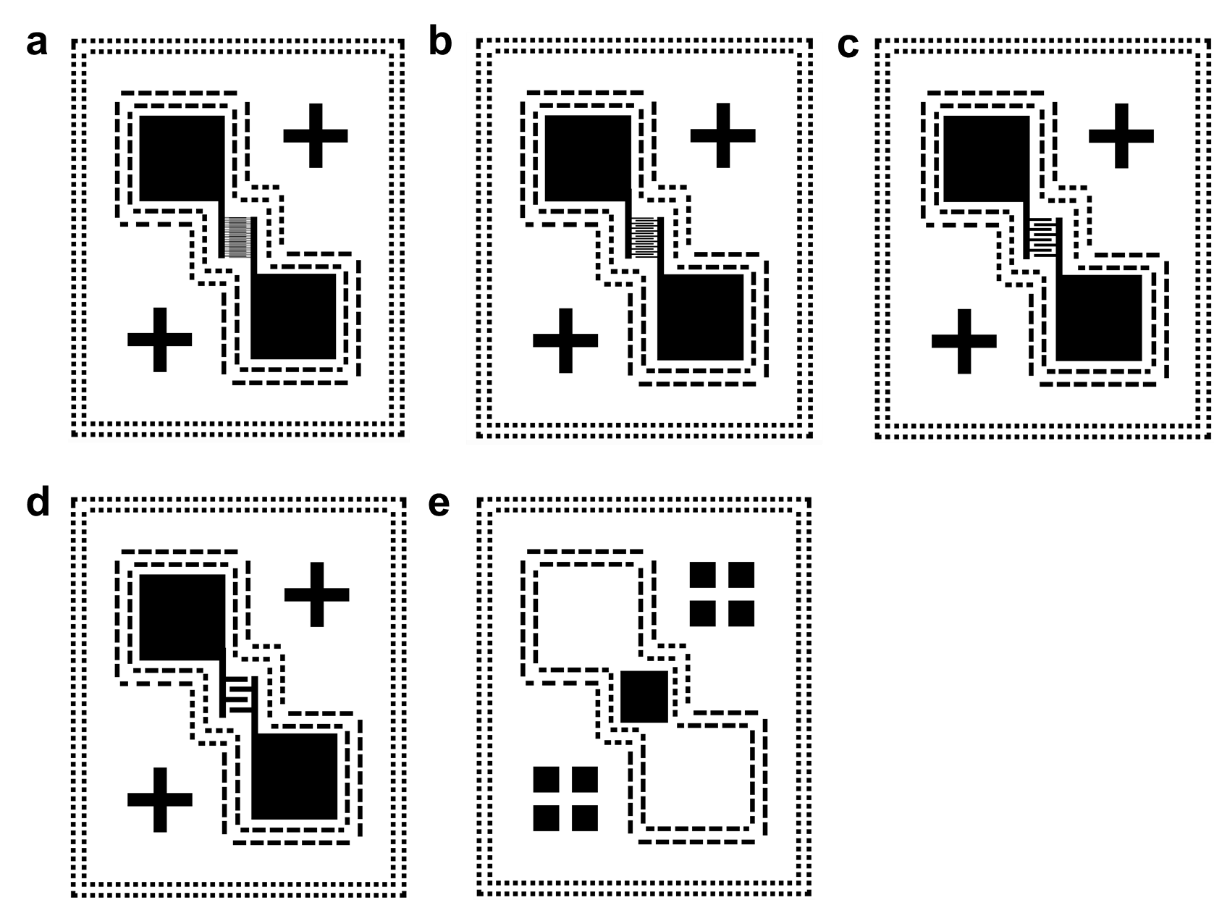


**Fig. S2 Layout design of electrode and ZnO alignment.** Layout designs of **a** 10-μm-design electrode, **b** 30-μm-design electrode, **c** 60-μm-design electrode, and **d** 120-μm-design electrode. **e** Layout design for ZnO alignment patterning.


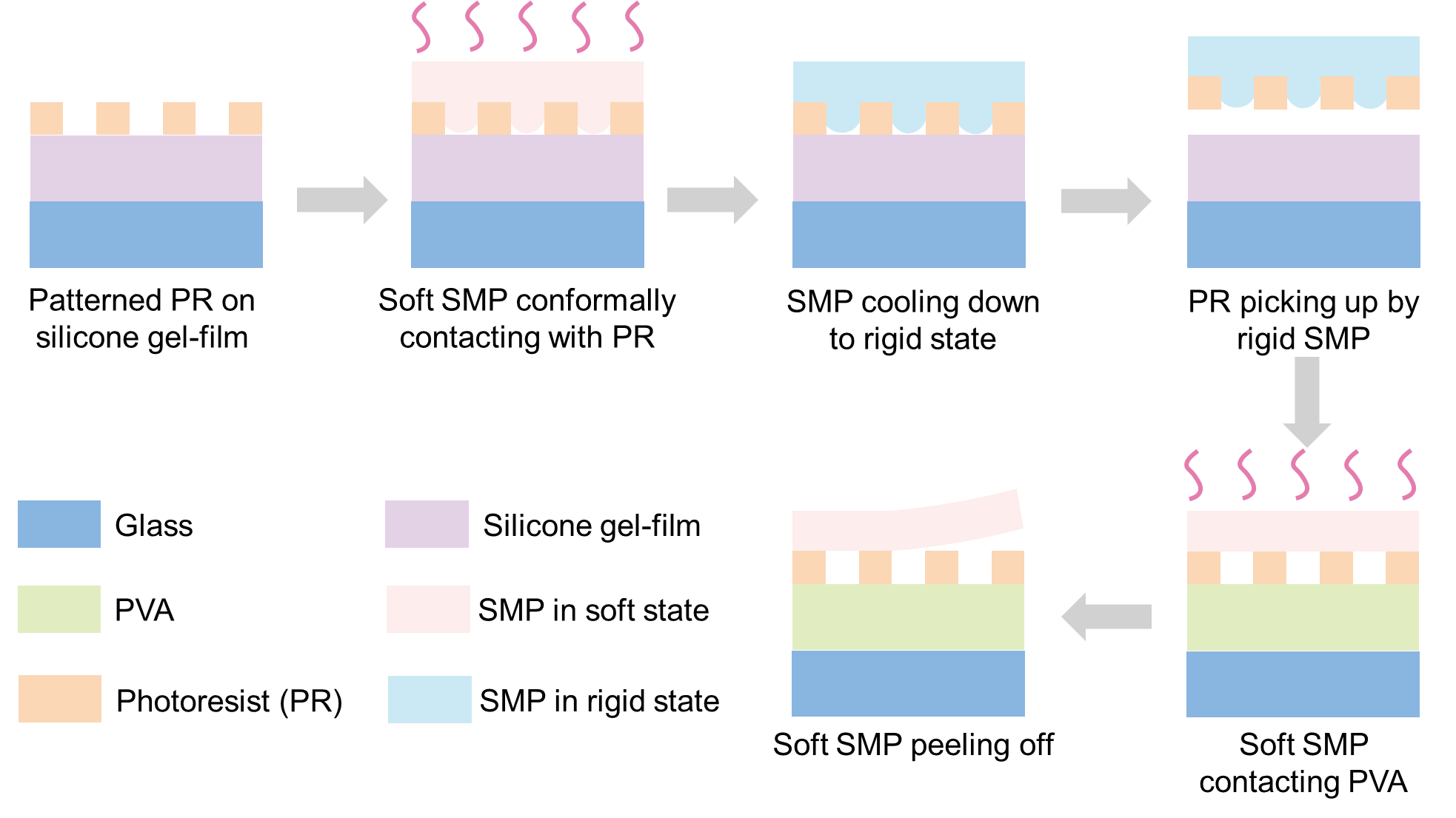


**Fig. S3 Schematic of PR transfer process.**


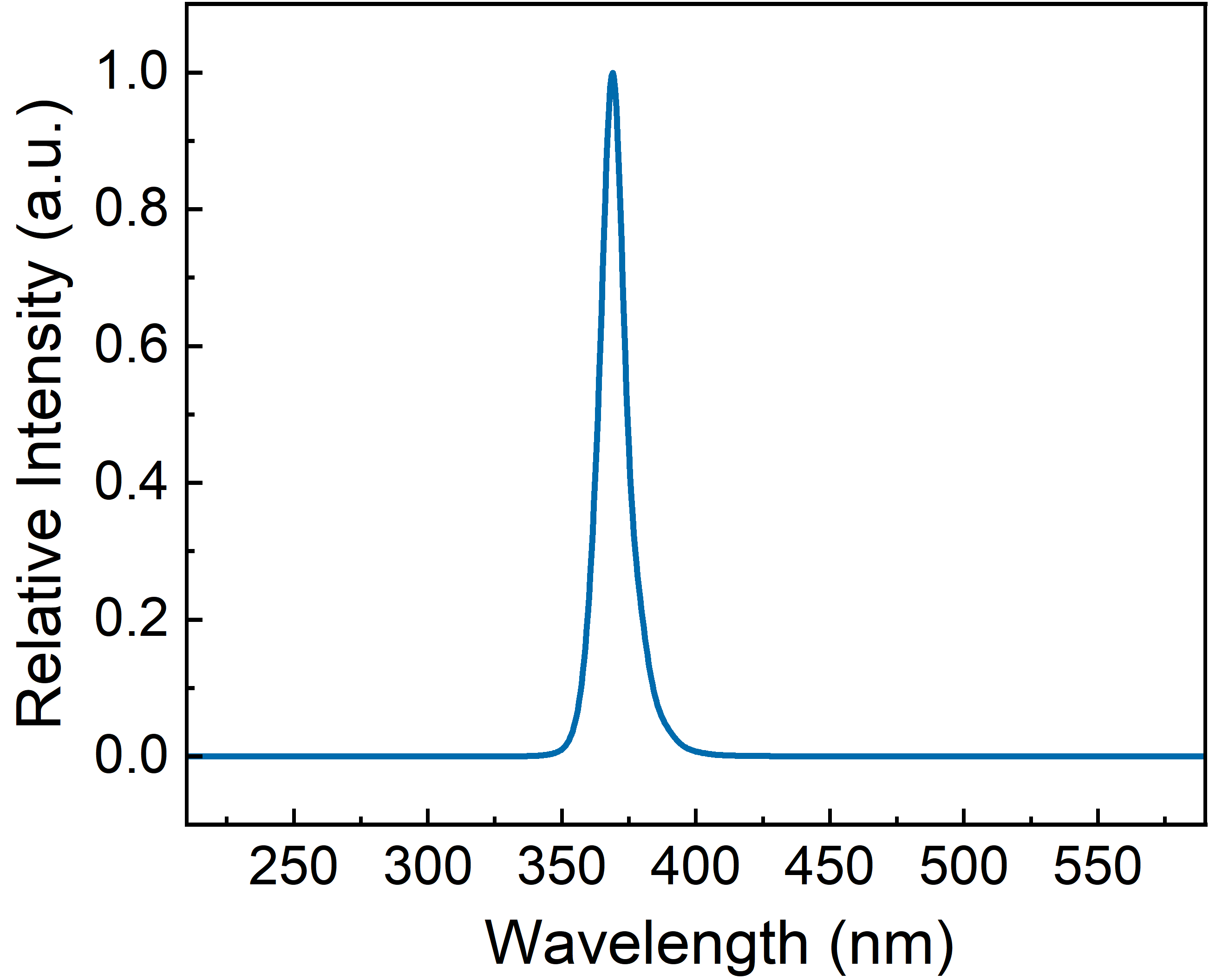


**Fig. S4 Emission spectrum of the UV source used in the experiment**

**Analysis on the effect of interdigital electrode linewidth on current**

As the mechanism of photodetector is based on photoelectron generation, the ZnO layer between electrode fingers can be considered as resistors. A simplified model is presented in Fig. S5a. The whole device can be simplified to several resistors connected in parallel, as presented in Fig. S5b. An individual resistor *R* is calculated by Eq. 1:

|  | (1) |
| --- | --- |

Where *d* is the overlapped length of a pair of interdigital fingers; *w* is spacing width and linewidth of interdigital finger, both of which are designed to be the same; is resistivity of semiconductor thin film; *t* is the thickness of semiconductor layer.

The total resistor of the electrode is calculated by Eq. 2:

|  | (2) |
| --- | --- |

Where *D* is the length of the interdigital electrode area. is the number of interdigital gaps.

In Eq. 2, when the electrode area is constant (*d* and *D* are constant) and for the same ZnO thin film ( and *t* are constant), smaller linewidth of interdigital fingers *w* leads to lower total resistor . It means that higher resolution of interdigital electrode provides larger and more detectable current.


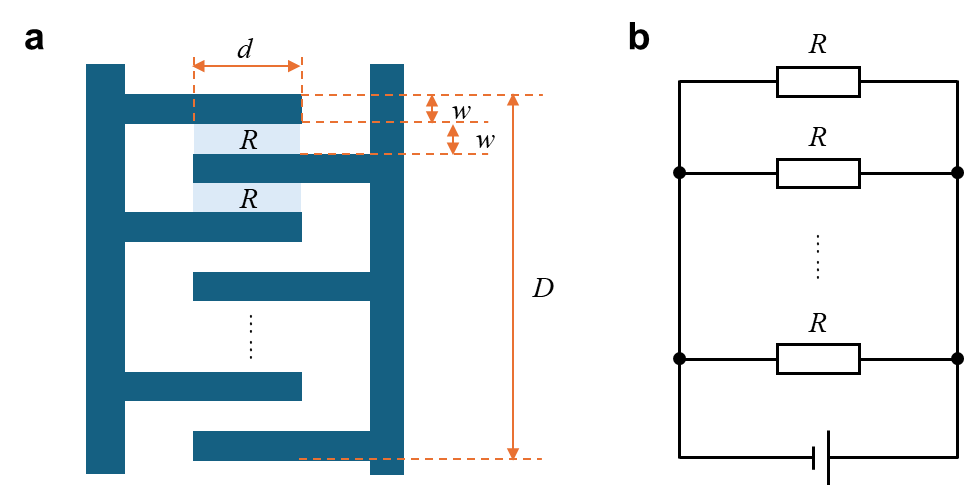


**Fig. S5 Simplified model of interdigital electrode. a** Schematic of interdigital electrode. **b** Equivalent circuit of the sensor with interdigital electrode.

REFERENCES

1. Q. Guo. *et al*. Micro-Transfer Printing of Photoresist Using Adhesion- Switchable Stamp for Patterning Unconventional Surface. 2024 IEEE 37th International Conference on Micro Electro Mechanical Systems (MEMS 2024), 669-672, Austin, TX, USA, Jan 21-25 (2024).
